# The hypoxia-selective cytotoxin NLCQ-1 (NSC 709257) controls metastatic disease when used as an adjuvant to radiotherapy

**DOI:** 10.1038/sj.bjc.6605753

**Published:** 2010-06-29

**Authors:** S J Lunt, C Cawthorne, M Ali, B A Telfer, M Babur, A Smigova, P J Julyan, P M Price, I J Stratford, W D Bloomer, M V Papadopoulou, K J Williams

**Affiliations:** 1 School of Pharmacy and Pharmaceutical Sciences, Stopford Building, University of Manchester, Oxford Road, Manchester M13 9PT, UK; 2 Academic Department of Radiation Oncology, The Christie NHS Foundation Trust, Manchester M20 4BX, UK; 3 Wolfson Molecular Imaging Centre, University of Manchester, Manchester M13 9PT, UK; 4 Department of Radiation Medicine, NorthShore University, HealthSystem, Evanston, IL 60201, USA

**Keywords:** NLCQ-1 (NSC 709257), bioreductive, hypoxia, radiotherapy, metastasis, PET

## Abstract

**Background:**

Metastases cause most cancer-related deaths. We investigated the use of hypoxia-selective cytotoxins as adjuvants to radiotherapy in the control of metastatic tumour growth.

**Methods:**

The NLCQ-1, RB6145 and tirapazamine were assessed against the spontaneously metastasising KHT model. Subcutaneous KHT tumours (250 mm^3^) were irradiated with 25 Gy (single fraction) to control primary growth. Equitoxic drug treatments (NLCQ-1 (10 mg kg^–1^) once daily; RB6145 (75 mg kg^–1^) and tirapazamine (13 mg kg^–1^) twice daily) were administered 3–6 days post-radiotherapy when hypoxic cells were evident in lung micrometastases. Mice were culled when 50% of controls exhibited detrimental signs of lung metastases.

**Results:**

In total, 95% of control mice presented with lung disease. This was significantly reduced by NLCQ-1 (33% *P*=0.0002) and RB6145 (60% *P*=0.02). Semi-quantitative grading of lung disease revealed a significant improvement with all treatments, with NLCQ-1 proving most efficacious (median grades: control, 4; NLCQ, 0 (*P*<0.0001); RB6145, 1 (*P*<0.001), tirapazamine, 3 (*P*=0.007)). Positron emission tomography (PET) was evaluated as a non-invasive means of assessing metastatic development. Primary and metastatic KHT tumours showed robust uptake of [^18^F]fluorodeoxyglucose ([^18^F]FDG). Metastatic burden discernable by [^18^F]FDG PET correlated well with macroscopic and histological lung analysis.

**Conclusion:**

The hypoxia-selective cytotoxin NLCQ-1 controls metastatic disease and may be a successful adjuvant to radiotherapy in the clinical setting.

Hypoxia is a common physiological abnormality in solid tumours and is known to be associated with resistance to conventional therapeutics. Further, there is a large body of evidence to suggest that tumour hypoxia is linked to metastatic progression (Lunt *et al*, 2009). These data, together with the low incidence of hypoxia in physiologically normal tissues, mark tumour hypoxia as an attractive and exploitable therapeutic target. Compounds known as bioreductive drugs or hypoxia-selective cytotoxins have been developed. These are inert pro-drugs that are activated by reductive enzymes in a hypoxic environment generating toxic metabolites that cause cell damage and death by various mechanisms (Stratford and Workman, 1998; McKeown *et al*, 2007). Ordinarily, hypoxia-selective cytotoxins are used in combination with other treatment strategies that preferentially target rapidly proliferating cells and/or whose efficacy is limited in the absence of oxygen. Synergistic interactions are often observed in this setting (Stratford *et al*, 2003).

Given the experimental and clinical links between hypoxia and metastases, we earlier hypothesised that selective targeting of hypoxic cells before radiotherapy would reduce the propensity for metastases. Indeed, we showed that the hypoxic cytotoxin tirapazamine reduces metastatic dissemination from hypoxic primary tumours when it is given before radiotherapy (Lunt *et al*, 2005). This could suggest that either the hypoxic cells are the population most likely to metastasise or that hypoxic cell signalling influences dissemination.

An alternative way in which hypoxia-selective cytotoxins could be exploited in an anti-metastatic strategy is based on the assumption that early stage metastases will contain hypoxic cell foci, as has been recognised for a number of years (Stanley *et al*, 1977). These could have an important function in the induction of an ‘angiogenic switch’ as hypoxia is an important physiological trigger for the increased expression of a number of angiogenic cytokines and their receptors including vascular endothelial growth factor (Semenza, 2009).

The aim of this study was to ascertain whether hypoxia-selective cytotoxins could be given as adjuvants to radiotherapy to control metastases. This has been analysed using the KHT murine sarcoma established in its syngeneic host (C3H mouse). The KHT model was used as it develops spontaneous lung metastases after primary subcutaneous tumours are controlled with radiotherapy (Baker *et al*, 1981; Lunt *et al*, 2005). The model is very aggressive, with metastases forming in a reproducible temporal and spatial manner allowing robust evaluation of anti-metastatic strategies. Three different hypoxia-selective cytotoxins were used: tirapazamine, RB6145 (less emetic bromoethyl precursor of the bioreductive RSU1069) and one of the newer generations of bioreductive compounds, NLCQ-1 (NSC 709257). These drugs differ in terms of the oxygen concentration required for cytotoxicity. Both RB6145 and NLCQ-1 are nitroimidazoles that require oxygen levels below 0.1% for maximal bioactivation (Koch, 1993; K Williams and MV Papadopoulou, unpublished data). Tirapazamine becomes increasingly cytotoxic as oxygen levels decrease (Koch, 1993). Recent work has suggested that the metabolism of tirapazamine at intermediate oxygen tensions may compromise the penetration of the drug into hypoxic regions (Kyle and Minchinton, 1999; Hicks *et al*, 2006). Indeed, NLCQ-1 was rationally designed to specifically possess weak DNA affinity to allow for good extra-vascular diffusion and penetration to hypoxic tumour tissue. The NLCQ-1 enhances the effect of radiotherapy, radioimmunotherapy and chemotherapy and compares favourably with tirapazamine when assessed in head-to-head studies (Papadopoulou and Bloomer, 2003). Here, we show that the nitroimidazoles and in particular NLCQ-1 significantly inhibit metastatic growth when administered post-radiotherapy. Further, we have investigated the use of positron emission tomography (PET) as a clinically relevant imaging tool to assess metastatic burden. Using this non-invasive technique, we found that KHT lung metastases are avid by [^18^F]fluorodeoxyglucose ([^18^F]FDG) PET and that lung burden assessed through imaging equated well with alternative assessment methods. Given that metastases are the most common reason for mortality in cancer patients, these findings have marked clinical relevance and support the use of hypoxia-selective chemotherapy in the adjuvant setting.

## Materials and methods

### Cell culture

KHT cell cultures were established from viably frozen stock tumour pieces. These were rapidly thawed to 37°C, freeze medium removed and the tumour piece cross-chopped in a small volume of RPMI medium supplemented with 10% foetal calf serum and 2 mM glutamine (RPMI complete). The resultant brie was then repeatedly pippeted to disaggregate remaining cell clumps and the suspension plated into a standard culture flask. All *in vivo* experiments were undertaken using cells established from the same KHT stock tumour after minimal *in vitro* passaging (<6 passages).

### Proliferation assay

KHT cells were seeded at 500 cells per well into 96-well plates. Cells were left 2–3 h to attach. NLCQ-1 was prepared at a range of concentrations by serial dilution and added to the cells for 3 h. For anoxic exposure, cell seeding and drug preparation were undertaken in an anoxic chamber (Bactron anaerobic chamber, Sheldon Manufacturing, OR, USA) using plastics that had been held within the anoxic chamber for at least 3 days and medium that had similarly been primed to the hypoxic condition. The drug-containing medium was then replaced with fresh culture medium. Cells were cultured for 4 days under standard conditions (95% air, 5% CO_2_, humidified). The MTT (3-[4,5-dimethylthiazol-2-yl]-2,5 diphenytetrazolium bromide) was then added to a final concentration of 5 mg ml^–1^ for 4 h. Medium was then removed and the cells lysed in dimethylsulphoxide. The optical density (OD) of the formazan product was read at 540 nm using a 96-well plate reader (BioTek *μ*Quant, Bedfordshire, UK).

### *In vivo* studies

The tumour studies analysing spontaneous metastases formation were performed as earlier described in Lunt *et al* (2005). Briefly, 5 × 10^5^ KHT cells prepared in a 0.1 ml volume of serum-free RPMI were implanted subcutaneously on the back of female C3H mice (age 10–12 weeks). Tumour volume measurements were initiated once a palpable tumour formed. Tumour localised radiotherapy (25 Gy) was administered under ambient conditions to non-anaesthetised mice-bearing tumours of 200–300 mm^3^ in size that were restrained in lead shielded containers. Treatment with bioreductive agents was initiated 72 h after radiotherapy in combination studies. Drugs were all given by intra-peritoneal (IP) injection in fractionated protocols over a 4-day period. Doses were as follows: NLCQ-1, 15 mg kg^−1^; RB6145, 75 mg kg^−1^; tirapazamine, 13 mg kg^−1^. The NLCQ-1 was given once daily and RB6145 and tirapazamine twice daily at 12 h intervals. These were equitoxic dosing regimens for the three compounds. Control mice received 0.9% saline. For metastasis experiments, external evidence of lung metastases in 50% of control mice (weight loss and/or laboured breathing) indicated, from experience, that the experimental end point had been reached. In addition, subsets of tumour-bearing mice were killed 1, 3 and 5 days post-radiotherapy or 16 h after the completion of the treatment with bioreductive drugs. In all treatment groups, pimonidazole (60 mg kg^−1^ IP; Chemicon International Inc., CA, USA) was given 2 h before killing. Lungs and residual primary tumours were rapidly excised and either snap frozen in liquid nitrogen or formalin fixed for histological analysis. As an alternative method for generating lung ‘metastases’, KHT cells were administered by tail-vein injection (5 × 10^4^ cells in 0.1 ml per mouse). Fractionated drug treatments commenced 72 h after cell implant. Mouse welfare was monitored at least daily and more frequently when the experimental end point was approached. All procedures were carried out in accordance with the Scientific Procedures Act 1986 and UKCCCR Guidelines 1997 by approved protocols (Home Office Project Licences 40-1770 and 40-2328 held by IJS and 40/3212 held by KJW) following institutional guidelines.

### Analysis of local control

Primary tumour volumes were monitored post-therapy. Local control was documented when there was no evidence of primary tumour at the point of killing. Local treatment failure or evidence of re-growth described either tumours, which showed little response to the local therapy (i.e., did not regress) or tumours, which initially regressed (often to a point where there was no evidence of tumour), but then re-lapsed and were palpable at killing.

### Scoring of metastatic burden

After treatment, lungs were rapidly excised and fixed in Bouin's solution (2.5% in 0.9% saline; Sigma Diagnostics, Gillingham, UK) to easily visualise tumour deposits. The severity of metastases was assessed using a semi-quantitative scoring system that has been described earlier (Lunt *et al*, 2005). Briefly, the lungs were awarded a grade from 0 to 5 depending on the extent of disease with a ‘0’ score indicating no visible metastases and ‘5’ indicating >80% of the lung surface was diseased (Lunt *et al*, 2005).

### *Ex vivo* clonogenic assay of tumour cell viability

When the potential onset of metastatic disease was apparent in control mice, mice receiving radiotherapy plus/minus NLCQ-1 were culled and lungs were rapidly excised. They were weighed and an assessment of metastatic burden made according to the criteria listed above. The lungs were cross-chopped in a small volume of RPMI complete, supplemented with penicillin and streptomycin (RPMI P/S). Additional medium was added and the lung brie passed through a sterile 35 *μ*m mesh. The cell suspension was pelleted, resuspended in phosphate-buffered saline (PBS), pelleted again and then resuspended in RPMI P/S giving a final preparation equating to 0.1 g lung weight 1 ml^−1^ medium. This suspension was serially diluted and each dilution plated in duplicate into six-well plates. Colony formation was assessed 10 days later.

### Positron emission tomography

Tumour studies for PET were carried out as described above. For the pilot study, animals underwent dynamic [^18^F]FDG scanning ∼9 days after cell implant when xenograft size had reached 200–250 mm^3^. Animals were fasted overnight before scanning, staggered across the group to reflect scanning times. Animals were anaesthetised with 1–2% isoflurane and injected with ∼10 MBq [^18^F]FDG (Erigal, Keele, UK) intravenously (IV) through the tail vein. They were then scanned on the quad-high-density avalanche chamber (HIDAC) system (Oxford Positron Systems, Weston-on-the-green, UK) with a theoretical resolution of 1 mm (Hastings *et al*, 2007). List mode data were collected for 75 min with anaesthesia being maintained during image acquisition through a nose cone on the scanning rig, with respiration monitored throughout using a pressure sensitive pad (Model 1025L, SA Instruments, NJ, USA). Body temperature was monitored throughout using an infrared thermometer and maintained by use of a heating pad, a hot air blower and a heating lamp. Animals were recovered in a warmed cage. For the main study, animals underwent static [^18^F]FDG scanning at 13 and 20 days post-irradiation. The procedure was identical to dynamic scanning except that the animals were anaesthetised for 45 min on the bench before being scanned for a further 15 min. Immediately after the last scan, tumours were excised and fixed in 10% formalin. Pilot [^18^F]fluoro-L-thymidine ([^18^F]FLT) studies were carried out as described above, with animals imaged under anaesthesia for 60 min after injection with 10 MBq [^18^F]FLT. [^18^F]FLT was synthesised ‘in house’ using published protocols (Blocher *et al*, 2002). Images were reconstructed using OPL-EM (Reader *et al*, 2002). Absolute calibration of the images was achieved by reference to a ^22^Na source imaged in the field of view in each scan. This had been validated with a uniformly filled mouse-sized [^18^F] phantom imaged over 2 h. Static images were constructed and uptake values calculated for the region of interest (ROI) expressed as kBq ml^–1^. The ROI analysis was undertaken using in-house developed software. Further normalisation was performed using the injected dose (from the dose calibrator) and animal weight to give a standardised uptake value (SUV); SUVmax was calculated from the maximum voxel value within the ROI and SUVmean as the average overall voxels. For [^18^F]FDG, normalised uptake values (NUVs) were calculated by dividing SUVmax or SUVmean from the tumour by that from the brain.

### Immunohistochemistry

#### Lungs

In total, 5 *μ*m sections were taken from formalin-fixed paraffin-embedded samples. Pimonidazole adduct formation was revealed using Hypoxyprobe-1 monoclonal antibody (Chemicon International Inc.) and the mouse Envision kit (Dako Ltd., Ely, UK) according to the manufacturer's guidelines for both reagents. Sections were counterstained using Gill's haematoxylin. Haemotoxylin and eosin (H&E) staining was undertaken on sequential sections for comparison.

#### Primary tumours

Cryostat sections (5 *μ*m) were prepared from snap-frozen tumour material and fixed using ice-cold acetone. Sections were simultaneously treated with rat anti-CD31 (1 : 250 dilution; Pharmingen, BD Biosciences, Oxford, UK) and Hypoxyprobe antibody (1 : 50 dilution in PBS supplemented with 0.1% bovine serum albumin and 0.1% Tween) followed by TRITC-labelled goat anti-rat (Molecular Probes, Invitrogen Life Technologies, Paisley, UK) and rabbit anti-mouse FITC (Dako) antibodies. Vessel density and hypoxic fractions were analysed using a NIKON Eclipse E800 with associated MetaMorph software. Vessel density was analysed per unit area of the tumour section and hypoxia as the fraction positive area as described earlier (Williams *et al*, 2007).

### Data analysis

Presence or absence of metastases was assessed by Fisher's exact test using the online VasserStats package. Local control data was analysed using Kaplan–Meier plots and log-rank *P*-values determined using Origin. Differences in the frequency distributions of metastatic burden were assessed by Mann–Whitney *U-*tests using Origin.

## Results

### Hypoxic KHT cells are sensitive to NLCQ-1 *in vitro*, but NLCQ-1 shows no single agent activity against KHT tumours *in vivo*

**Figure 1:**
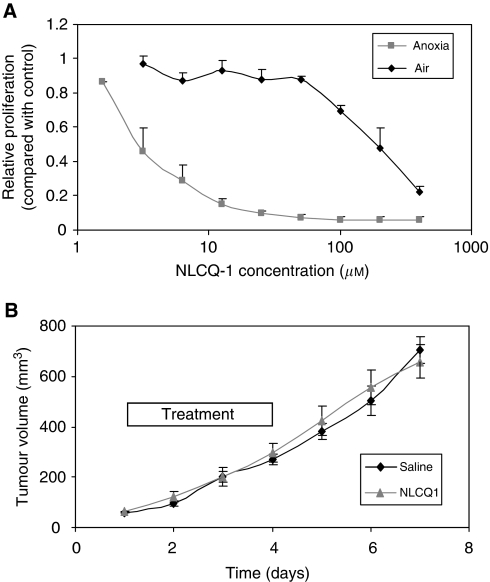
NLCQ-1 is an effective hypoxic cytotoxin against KHT cells *in vitro*, but does not impair KHT tumour growth *in vivo*. KHT cells (**A**) were exposed to NLCQ-1 under aerobic or anoxic conditions for 3 h. Proliferation was assessed 4 days later by MTT assay. Data points represent the average relative proliferation compared with untreated control cells from three experiments (±s.d.). Exposure to anoxia alone did not influence KHT proliferation (average OD_540_ aerobic controls 1.4 *vs* 1.3 for anoxic controls). (**B**) KHT cells were inoculated into C3H mice. Once tumours had established NLCQ-1 was administered daily for 4 consecutive days (15 mg kg^–1^). Presented are average tumour volume measurements (±s.e.; *n*=8 per group).

The hypoxia selectivity of tirapazamine and RSU1069 in the KHT model has been established earlier (Stratford *et al*, 1986; Siemann, 1990; Stratford, 1992). Here, initial studies were undertaken to assess the relative sensitivity of KHT cells to NLCQ-1. Cells were exposed to NLCQ-1 for 3 h under aerobic or anoxic conditions and proliferation relative to untreated controls assessed 4 days later. The NLCQ-1 was markedly more toxic against KHT cells exposed under anoxic *vs* aerobic conditions (Figure 1A). The NLCQ-1 concentration required to inhibit proliferation by 50% was 202 *μ*M±32 (s.e.) in air compared with 2.9 *μ*M±1.0 (s.e.) under anoxic conditions giving a hypoxic selectivity ratio of ∼70-fold. To assess whether the cytotoxic effect of NLCQ-1 would translate into efficacy as a single agent, KHT tumours were established in C3H mice and treated with fractionated NLCQ-1 treatment (15 mg kg^−1^ daily for 4 days). Consistent with our earlier published observations with the bioreductive agents RB6145 and tirapazamine (Lunt *et al*, 2005), NLCQ-1 treatment alone has no effect on the growth of primary KHT tumours (Figure 1B). Earlier pharmacokinetic studies showed that the peak plasma concentration of NLCQ-1 after a 10 mg kg^–1^ bolus was 26.8 *μ*M at 5 min post-administration (Reid *et al*, 2003). This suggests that the dose used in this study would be sufficient to target hypoxic, but not oxic KHT cells within the tumour mass. The lack of effect of NLCQ-1 as a single agent suggests that overall tumour growth is dominated by the oxic population.

### Early stage KHT lung micrometastases contain hypoxic cells

**Figure 2:**
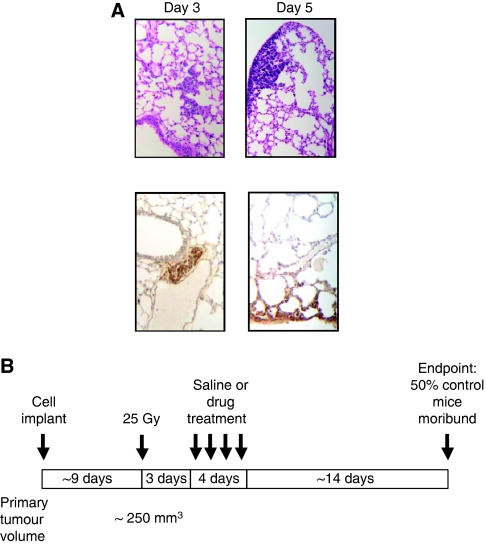
Hypoxic KHT lung micrometastases are evident 3 days after radiotherapy of the primary tumours. (**A**) Primary KHT tumours were irradiated with 25 Gy and lungs excised at various times thereafter. Pimonidazole was administered before lung excision and adduct formation (brown staining) revealed after immunohistochemical analysis (top panels). The H&E staining in comparative sections is shown in the lower panels. (**B**) Schematic of treatment protocol.

The aim of these studies was to investigate the function of bioreductive drugs as adjuvants to radiotherapy in the control of metastatic disease. Initial experiments were undertaken to examine whether KHT lung micrometastases contained a target hypoxic cell population. Primary KHT tumours were established and irradiated with 25 Gy. The hypoxic marker pimonidazole was administered and lungs excised at various times post-radiotherapy. Micrometastases were not clearly evident 1-day post-radiotherapy (not shown). However, by day 3 metastatic deposits that stained positively for pimonidazole adducts were revealed after immunohistochemical analysis (Figure 2A).

### NLCQ-1 and RB6145 have significant activity against spontaneously arising KHT metastases in the lung

**Figure 3:**
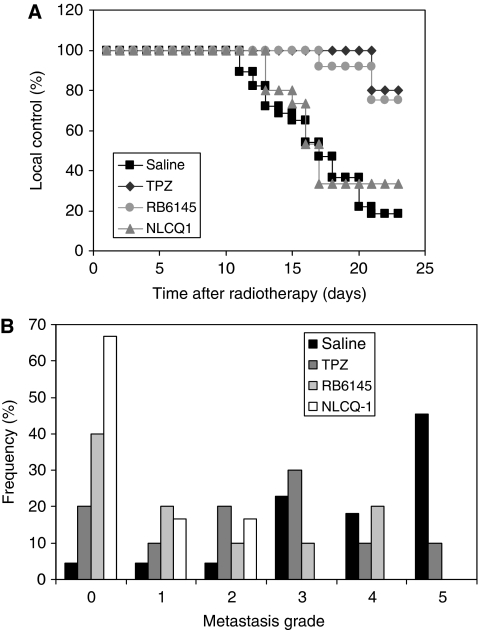
NLCQ-1 yields little improvement in local control compared with tirapazamine and RB6145, but is superior in the control of metastatic disease when administered as an adjuvant to radiotherapy. Primary tumour response was monitored post-treatment with radiotherapy (administered on day 1) and bioreductive agent or saline (days 4–7) (**A**). Evidence of re-growth after treatment (see Materials and Methods for details) was taken as a loss of local control and this is expressed as per cent of treated mice with time after treatment. KHT lung metastatic burden was assessed using a semi-quantitative system when 50% of saline-treated control mice exhibited evidence of disease (**B**). The histograms show the frequency distribution of metastatic grade for each treatment. Data shown were pooled from three independent experiments. The first compared RB6145 (*n*=10) and tirapazamine (*n*=10) *vs* saline control (*n*=12); the second NLCQ-1 (*n*=9) *vs* saline control (*n*=7) and the third NLCQ-1 *vs* saline control (*n*=3/group). There was no significant difference in the control data for the three experiments. Bioreductive treatments were NLCQ-1, 15 mg kg^–1^ once daily; tirapazamine 13 mg kg^−1^ twice daily; RB6145 75 mg kg^−1^ twice daily or saline twice daily.

To assess the effects of bioreductive drugs on KHT lung metastases, treatment was initiated 3 days after radiotherapy of primary tumours and continued for a 4-day period (see Figure 2B for a schematic of the treatment schedule). Both primary tumour growth and the onset of metastases (evidenced by loss of condition) were monitored in treated and control populations. Three experiments were undertaken and the data pooled in Figure 3 (see figure legend for details). In each experiment, there was no significant difference in the metastatic profile observed in saline-treated control tumours.

In terms of local control, NLCQ-1 yielded some improvement over saline (33% *vs* 18%), but this was not significant (log-rank *P* 0.5). However, both tirapazamine and RB6145 were far superior (Figure 3A) and had a highly significant impact on local control with >75% of treated mice showing no evidence of primary tumour re-growth during the course of the experiment (log-rank *P*<0.001 for both agents).

In contrast with the effects on primary tumour burden, NLCQ-1 proved most efficacious against metastatic disease. When 50% of control saline-treated mice showed evidence of metastases, all mice were killed and lung tumour burden assessed using an earlier established semi-quantitative scoring index (Lunt *et al*, 2005). Eight of 12 NLCQ-1-treated mice had no evidence of macroscopic disease (Figure 3B). None of the NLCQ-1-treated mice presented with greater than grade 2 disease and the median metastasis score was 0. The RB6145 also increased the frequency of lung disease-free mice compared with controls (40% *vs* 5%). Here, the median grade was 1. Somewhat surprisingly, tirapazamine was the least effective of the agents assessed with a median lung score of 2.5 observed after treatment and only 20% of the mice appear free from lung tumours. High-grade lung disease was observed in a significant proportion of treated mice, with 50% of mice presenting with grade 3 disease or above. Comparing the data distributions by Mann–Whitney *U*-test supported the significance of these qualitative observations (*P*-values for NLCQ-1, RB6145 and tirapazamine *vs* saline-treated controls were <0.00001, <0.001 and 0.007). In the clinical scenario, a single metastatic lesion can be lethal; therefore, data were also analysed using a binary score for the presence or absence of metastases. Again the impact of NLCQ-1 was highly significant (*P*=0.0002 *vs* controls). The RB6145 also yielded a significant improvement (*P*=0.02), but this was not the case for tirapazamine (*P*=0.22; Fisher's Exact tests).

To complement the studies into effects against metastases that spontaneously arise from subcutaneous KHT tumours, experiments were undertaken to assess the impact of NLCQ-1 and tirapazamine treatment on lung metastases formation after intravenous injection of KHT tumour cells (experimental metastases). Initial studies were undertaken to determine the injected cell number required to give metastases over the same time frame as the interval between radiation and morbidity in the spontaneous model. In addition, histology was undertaken to ensure that metastases were present over the course of bioreductive treatment (days 3–6 post-cell injection inclusive; data not shown). Neither NLCQ-1 nor tirapazamine caused a significant change in the distribution of metastastic grade observed compared with saline-treated controls (*P*=0.12 and0.19 for tirapazamine and NLCQ-1, respectively, *n*=6 per group [data not shown]).

### NLCQ-1 treatment does not seem to be anti-angiogenic

Recent data have suggested that some bioreductive agents may have anti-angiogenic effects (Huxham *et al*, 2006; O'Rourke *et al*, 2008). An anti-angiogenic effect against the primary tumour could have conceivably influenced metastatic dissemination post-radiotherapy in the current studies. To investigate this, vessel density and hypoxic fraction were assessed in irradiated tumours after treatment with saline or NLCQ-1. The analysis was undertaken on tumours excised 16 h after the final saline/NLCQ-1 dose (*n*=4 per group). The KHT model is highly vascularised and control tumours had a vessel density of 86±13 (s.e.) mm^−2^. The NLCQ-1 reduced this slightly to 67±17 (s.e.) mm^−2^, but the change was not significant (*P*=0.48). Concomitant with the reduction in vessel density, irradiated NLCQ-1-treated tumours exhibited an increase in hypoxic fraction compared with saline-treated controls (13±3 *vs* 9±1 in controls), but again this did not achieve significance (*P*>0.05).

### Utilisation of non-invasive PET imaging to assess KHT lung metastases

**Figure 4:**
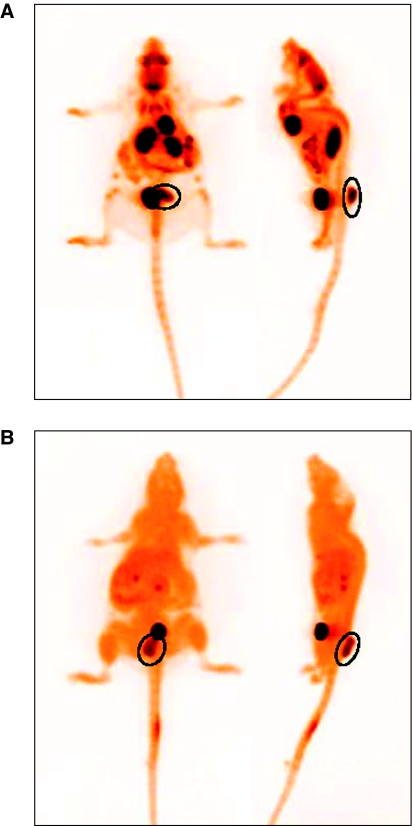
Primary KHT tumours (circled) show robust uptake of both [^18^F]FDG and [^18^F]FLT. Coronal and sagittal maximum intensity projections are shown, obtained 9 days after KHT cell implant after IV administration of ∼10 MBq [^18^F]FDG (**A**) or [^18^F]FLT (**B**).

**Figure 5:**
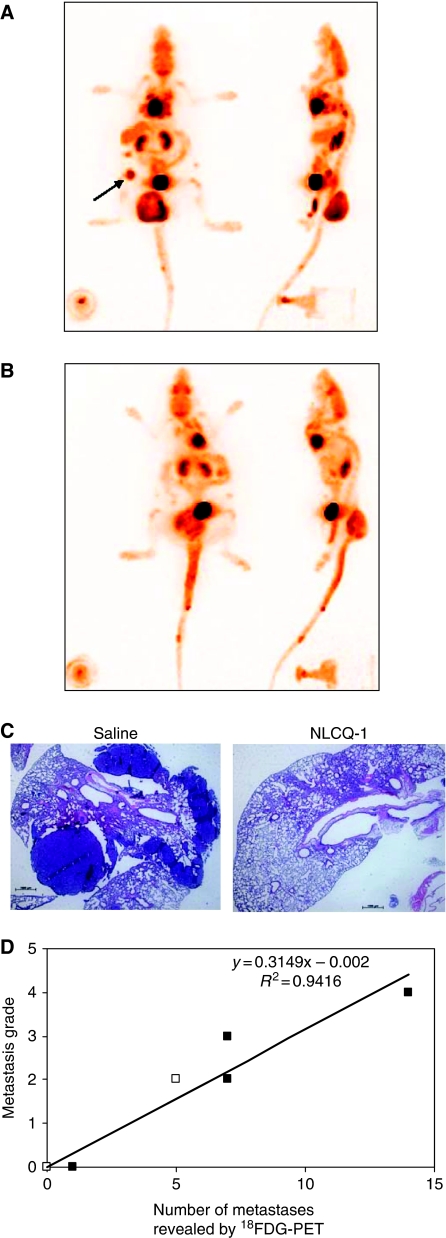
KHT lung metastases can be resolved using [^18^F]FDG PET. Coronal and sagittal maximum intensity projections are shown, obtained 20 days after primary tumour radiotherapy and subsequent treatment with saline (**A**) or NLCQ-1 (**B**) after IV administration of ∼10 MBq [^18^F]FDG. Note multiple lung metastases in (**A**) and the presence of a lymph node metastasis (arrow). H&E assessment of the lungs of the mice shown in (**A**) and (**B**) confirms the presence of lung metastases in the saline-treated mouse (**C**). The number of metastases resolved by PET shows an excellent correlation with the semi-quantitative scoring of burden in excised lungs (**D**). Closed symbols, control tumours; open symbols, NLCQ-1-treated tumours.

The experimental end point used in the preceding studies was defined by a loss of condition in the control group that occurred 3–4 weeks post-radiotherapy. The ability to image metastatic burden non-invasively using PET was investigated. Preliminary studies were undertaken using treatment-sized subcutaneous KHT tumours. Tumour-bearing mice were administered [^18^F]FDG or [^18^F]FLT. Primary tumours showed strong uptake of both [^18^F]FDG and [^18^F]FLT (Figure 4). Once uptake had been confirmed in primary tumours, two further studies were undertaken focused on the use of [^18^F]FDG to give an assessment of overall tumour burden. The first investigated [^18^F]FDG PET as a means to resolve KHT lung metastases. The second incorporated NLCQ-1 treatment. In total, seven animals that received saline and three animals that received NLCQ-1 post-radiotherapy were imaged. The KHT lung metastases were clearly evident in saline-treated mice imaged 20 days post-radiotherapy, before the onset of detrimental side effects (Figure 5A), although metastases were not visible at day 13. In addition, there was evidence of lymph node metastases that had not been recognised earlier (Figure 5A, arrow). As observed in the efficacy studies, NLCQ-1 treatment was associated with a reduced metastatic burden compared with saline controls (Figure 5). The presence and absence of metastases in the mice shown in Figures 5A and B was confirmed by (H&E) analysis of lungs excised post-imaging (Figure 5C). The number of metastases resolved by [^18^F]FDG PET showed a highly significant correlation (*P*<0.001) with those determined using the semi-quantitative visual scoring method (Figure 5D).

To assess how well visible scoring and imaging correlated with the presence of viable KHT clonogens within the lung tissue, lungs were excised from three saline and three NLCQ-1-treated animals 21 days post-radiotherapy, disaggregated and plated for clonogenic survival assays. Grade 0 lungs showed ∼1 clonogen per mg of lung tissue, whereas lungs with visible signs of metastasis (grades 1–5) showed an average of 165±57 clonogens per mg. Tumour tracer uptake was normalised to that observed in the brain. There was no significant difference in NUVs between treated and untreated primary tumours or metastases. The NUVmax and NUV mean were 1.1±0.3 and 1.0±0.3 for untreated primary tumours and 1.0±0.4 and 0.9±0.2 for treated tumours. Comparative values for metastases were NUVmax 0.9±0.2 for both groups and NUVmean 1.4±0.2 and 1.3±0.3 for untreated and treated, respectively.

## Discussion

NLCQ-1 (NSC 709257) is the lead bioreductive agent from a rational approach to develop nitroaromatics with weak DNA-intercalating properties that may improve drug biodistribution (Papadopoulou and Bloomer, 2003). Earlier studies have established that NLCQ-1 has a good hypoxia-selectivity ratio and combines well with radiotherapy and a range of chemotherapy approaches *in vivo* (Papadopoulou *et al*, 2001a, 2001b, 2002, 2005, 2006, 2007). Here, we show that NLCQ-1 is an excellent and well-tolerated adjuvant to radiotherapy in the control of metastatic tumour growth.

The KHT tumour model is a highly aggressive, locally invasive sarcoma that spontaneously metastasises to the lungs. It is almost unique in the reproducibility of the temporal onset of lung metastases, making it an ideal system in which to study anti-metastatic approaches. Our hypothesis in this study was that early stage metastases have a reliance on hypoxia to initiate gene expression changes that precipitate their subsequent growth. In preliminary studies, we were able to show that early stage microscopic KHT lung foci can be hypoxic. Up to day 5 post-radiotherapy of the primary tumour, there was no evidence of vascularisation of the hypoxic micrometastases. On the basis of the presence of a target population in the metastases, bioreductive treatment was given over 4 days starting 3 days after radiotherapy of the primary tumour.

Both RB6145 and tirapazamine improved the local control of the primary tumour, whereas NLCQ-1 had little effect. However, NLCQ-1 was the most effective at controlling metastases. Improved local control could conceivably influence the dissemination of tumour cells from the primary site. However, the fact that NLCQ-1 has little impact on local control suggests an alternative mechanism of action. We would argue that a direct effect against the hypoxic metastases is a strong candidate. In an attempt to remove the potential influence of primary tumour effects, the efficacy of NLCQ-1 and tirapazamine against KHT lung metastases established after tail-vein injection was assessed. Neither agent significantly affected metastatic growth. The apparent differences in efficacy against spontaneous *vs* experimental metastases perhaps supports that NLCQ-1 is indeed targeting an early step in spontaneous metastasis. Further, it may suggest that there are pathophysiologic differences between the two models. This may be associated with potential route of dissemination, given that cells could distribute from a primary tumour through lymphatic or blood vessels, whereas tail-vein injection forces dissemination through the latter route. Interestingly, pathological assessment of lungs from the spontaneous groups suggested lymphatic dissemination (data not shown). This speculation is perhaps supported by the presence of lymph node involvement as identified by [^18^F]FDG PET.

In our earlier studies, tirapazamine given before radiotherapy was able to reduce subsequent metastases formation, whereas RB6145 showed little effect (Lunt *et al*, 2005). Similarly, NLCQ-1 was unable to reduce metastatic dissemination when given pre-radiotherapy (data not shown). We hypothesised that the hypoxic primary tumour cells that lay at intermediate oxygen concentrations may be the most important target population in this context as these would be removed by tirapazamine, but not RB6145 or NLCQ-1 that require more stringent hypoxia for bioactivation (Koch, 1993; K Williams and MV Papadopoulou, unpublished data). Further tirapazamine was subsequently shown to have anti-angiogenic properties (Huxham *et al*, 2006), which may have impacted on the differential response. In this study, tirapazamine performed less well than the nitroimidazoles when given after radiotherapy. One possible explanation is that the bioavailability of tirapazamine is poorer in the lung tumours, perhaps being compromised by metabolism in relatively ‘oxygenated’ regions and by the fact that the foci seem effectively avascular (Kyle and Minchinton, 1999; Hicks *et al*, 2006). As NLCQ-1 was specifically designed to have enhanced penetration compared with other non-nitroimidazole-based bioreductives (Papadopoulou and Bloomer, 2003), this perhaps explains its enhanced activity in the current studies and highlights that when hypoxia-selective cytotoxins are used in conjunction with conventional therapy, oxygen dependence of activity, bioavailability and precise mechanism of cytotoxicity should all be taken into account to achieve an optimal response.

Although the KHT is an optimal model in many respects, one issue is that it is not possible to readily establish genetically modified clonal populations from these cells. This negates the possibility of using such approaches as bio-luminescence as non-invasive approaches to monitor disseminated tumour growth. We investigated whether the lung metastases could be resolved using [^18^F]FDG PET. The KHT model both established subcutaneously and when disseminated to the lung showed excellent uptake of [^18^F]FDG. In addition, there was a highly significant correlation between the metastatic profile obtained through [^18^F]FDG PET and that after histological examination of the lungs after killing of the tumour-bearing mice. Importantly, lungs that scored zero also had a low percentage of viable cells as measured by clonogenic assay. In addition, they had either no or single metastases discernible by [^18^F]FDG PET. This indicates that there is not significant viable cell mass escaping detection with [^18^F]FDG PET. As was observed in the efficacy studies, NLCQ-1 seemed to reduce the metastases detected by [^18^F]FDG PET at 20 days post-radiotherapy. Although partial volume effects will lead to a lessening of metastatic SUVs (Soret *et al*, 2007), as the tumour sizes (where present) were comparable between groups, the effect will occur to a similar extent irrespective of treatment. Although SUVs were reduced in treated metastases, this difference disappeared when uptake was normalised to the brain. As brain uptake should be similar between conditions, this suggests that drug treatment may have some systemic effect on tracer uptake, although greater study numbers (including data on blood pool activity) would be needed to confirm this. Recently, published observations confirm the uptake of [^18^F]FDG in primary KHT tumours (Liu *et al*, 2009), and show good uptake of [^18^F]fluoroacetate and [^18^F]fluoromisonidazole in this model. Given that our data also show strong uptake of [^18^F]FLT in primary tumours, this suggests that this fast-growing KHT model has particular usage in longitudinal PET-based imaging studies to assess tumour response to a range of targeted therapies in an immunocompetent host.

Overall, we summarise that bioreductive agents such as NLCQ-1 could have potential usage as adjuvants to radiotherapy in the control of metastatic disease and that clinically applicable non-invasive PET imaging can be used to monitor drug efficacy in the KHT model. Earlier studies have shown that bioreductive strategies synergise not only with radiotherapy, but also commonly used chemo- and chemo-radiotherapy in which hypoxia limits the effectiveness of treatment (Papadopoulou *et al*, 2001a, 2002, 2006; Le *et al*, 2009). The addition of hypoxia-selective chemotherapy alongside conventionally used clinical cancer therapy offers an attractive approach to target secondary disease, which remains the main cause of death in cancer patients.
